# Severe infection by *Vibrio anguillarum* following a bite by a marine fish: a case report

**DOI:** 10.1080/22221751.2023.2204145

**Published:** 2023-05-05

**Authors:** Jing-Yue Hu, Xiao-Kai Zhang, Cheng-Qi Xin, Lei Zhang, Jian Kang, Ping Gong

**Affiliations:** a Department of Emergency, The First Affiliated Hospital of Dalian Medical University, Dalian, People’s Republic of China; b Stem Cell Clinical Research Center, The First Affiliated Hospital of Dalian Medical University, Dalian, People’s Republic of China; c Dalian Innovation Institute of Stem Cell and Precision Medicine, Dalian, People’s Republic of China; d Department of Emergency, Shenzhen People’s Hospital (The Second Clinical Medical College, Jinan University; The First Affiliated Hospital, Southern University of Science and Technology), Shenzhen, People’s Republic of China

**Keywords:** *Vibrio anguillarum*, marine fish bite, infection, amputation, sepsis

## Abstract

*Vibrio anguillarum* is a cause of vibriosis in marine fisheries worldwide, but only one previous study reported human pathogenicity of this species. Here, we report a 70-year-old man from Dalian, a coastal city in northeast China, who experienced a severe infection with *V. anguillarum* due to a bite on his left hand when handling hairtail, a marine fish. This patient had low immunity because of the long-term use of glucocorticoids due to nephrotic syndrome. Despite treatments consisting of a strong antibiotic, continuous veno-venous hemofiltration, debridement, and fasciotomy, his condition deteriorated and he died of septic shock and multiple organ dysfunction syndrome. His death might be partly due to the delayed amputation of the left forearm, because he seemed to get better for the first several days. This case report emphasizes the possibility of human infection by *V. anguillarum*, which is likely to be more lethal in immunocompromised individuals.

## Introduction

*Vibrio* species are Gram-negative bacteria that have a curved-rod morphology, although species in the *V. cholerae* clade can have variable morphology [1,2]. *Vibrio* species generally occur in marine and estuarine environments [3,4]. Several species of *Vibrio* are pathogenic to humans, and three species (*V. cholerae* serogroups O1 or O139, *V. parahaemolyticus*, and *V. vulnificus*) are known to be responsible for serious human infections [1]. Vibriosis in a wound can lead to severe tissue damage, and vibriosis due to consumption of undercooked or raw seafood or contact with seawater can lead to diarrhoea and other extra-intestinal symptoms [2,3]. In the USA, *Vibrio* infection is responsible for about 80,000 illnesses, 500 hospitalizations, and 100 deaths each year [5].

*V. anguillarum* is a causative agent of vibriosis in marine fisheries worldwide, and this species can grow and proliferate efficiently under conditions of environmental stress [6]. Heretofore, the only report of a *V. anguillarum-*associated human illness was in an immunocompromised 65-year-old female from Maine (USA) who had a skin and soft tissue infection of left lower leg, and subsequently died from septic shock and multiorgan failure in July 2017 [7]. Here, we report a case of a 70-year-old man from China who developed a severe infection from *V. anguillarum* due to a bite on his left hand when handling hairtail, a marine fish.

## Case report

**Figure 1.**
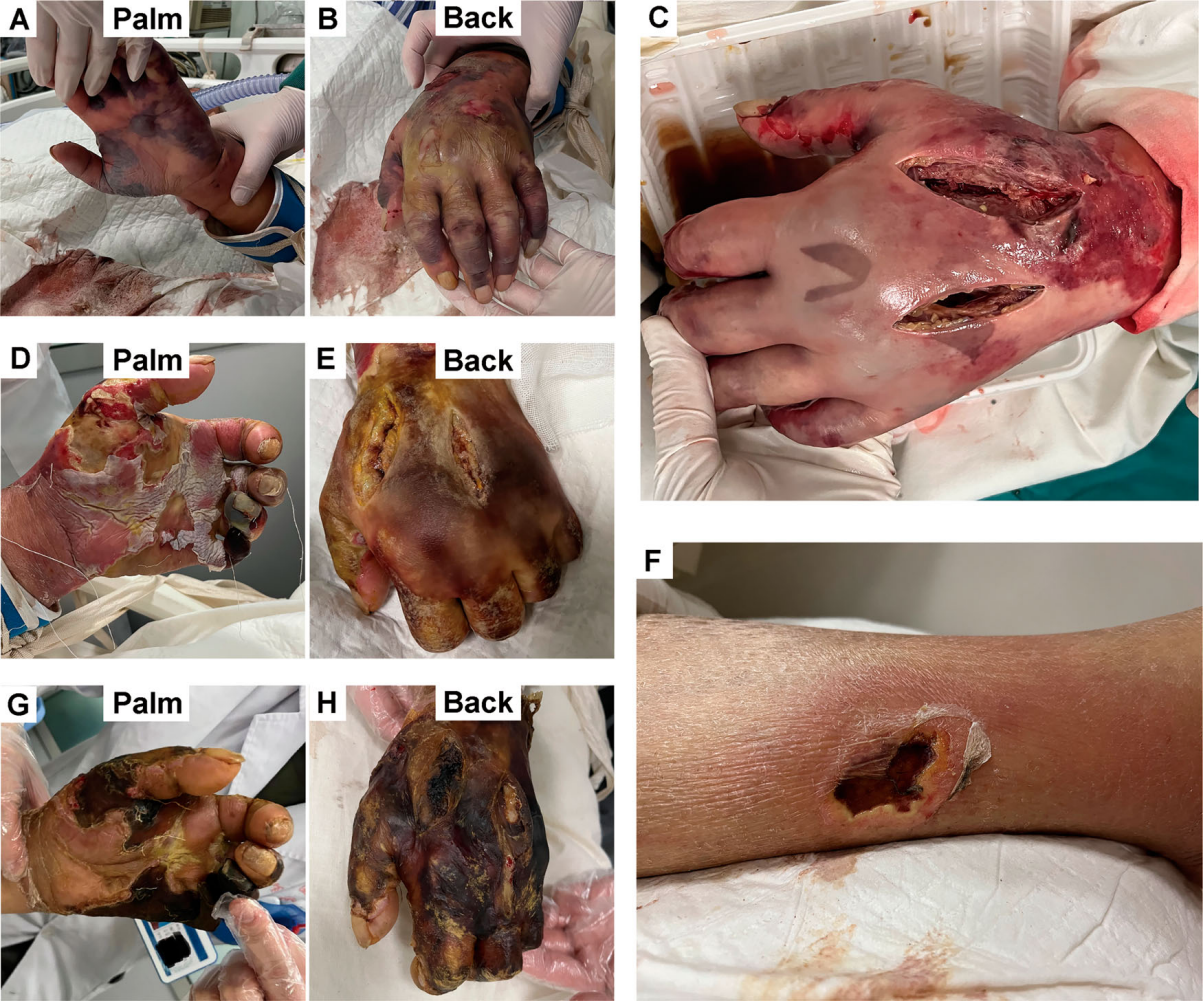
Changes of signs in the patient with severe *Vibrio anguillaris* infection. Three hours after admission, lesions of the left hand showed cyanosis, multiple blisters, and ecchymosis in the palm (A) and the back (B), with rupture of some large blisters. Three days after admission, debridement was performed with fascial compartment dissection for decompression (C). Ten days after admission, lesion of the left hand was deteriorated with the necrosis of the pinky finger (D and E). Fourteen days after admission, the left lower limb showed local lesion with local redness, partial ulceration, and bloody exudate (F). Twenty-six days after admission, necrosis of the left hand was uncontrolled (G and H).

The patient was a 70-year-old man who lived in Dalian, a coastal city in northeast China. He received a bite on his left hand when handling hairtail (a marine fish) on the morning of 10 October 2022, and developed persistent fever with redness and swelling of his left hand that afternoon. The next morning, he developed impaired consciousness and his relatives sent him to the emergency department of the First Affiliated Hospital of Dalian Medical University. At that time, his left hand was red and swollen, with blisters of different sizes (Figure 1(A,B). Some of the large blisters had ruptured, and the affected fingers could not flex or extend. The patient had a past history of hypertension, postoperative bladder cancer, and nephrotic syndrome, and was using oral glucocorticoids for about 6 months.

At admission, he presented with a body temperature of 38.7°C, blood pressure of 108/68 mmHg, heart rate of 130 bpm, and oxygen saturation of 98% when breathing ambient air. Preliminary blood tests showed significant elevations of procalcitonin (92.19 ng/mL), high sensitivity-troponin I (0.806 µg/L), B-type natriuretic peptide (854.57 ng/mL), and creatinine (157 µmol/L). At 1 h after admission, he experienced a drop in blood pressure to 72/38 mmol/L and an oxygen saturation of 92% (fraction of inspiration O_2_ [FiO_2_]: 3 L/min), and was diagnosed with septic shock. After administration of norepinephrine and fluid resuscitation, he was admitted to emergency intensive care unit (EICU). His SOFA score at that time was 7, indicating deterioration of parameters related to organ function, so he received urgent continuous veno-venous hemofiltration (CVVH). At 12 h after EICU admission, he received intratracheal intubation and mechanical ventilation due to dyspnoea and a decline of oxygen saturation (80%). A tracheotomy was administered 11 days later. Based on the diagnosis of sepsis and septic shock, the patient was given empirical antibiotic treatment with intravenous meropenem (1.0 g every 8 h). Before meropenem administration, samples of blood and blister fluid from his left-hand lesion were collected for metagenomics next-generation sequencing of pathogenic microbial DNA (Supplementary material). This sequencing was performed on a Nextseq 550 platform (Illumina, San Diego, USA) using a 50-cycle single-end sequencing strategy. Both results indicated *V. anguillarum* (Tables S1 and S2).

Three days after EICU admission, the lesion on his left hand had extended. Thus, debridement and fasciotomy were performed, with decompression and negative-pressure suction. After the surgery, the patient’s condition gradually improved, and there was reduced swelling and tension of the left hand (Figure 1(C)), restoration of consciousness, and a decreased level of procalcitonin and parameters indicative of organ dysfunction. Ten days after EICU admission, the hemofiltration was suspended, but there was evidence of necrosis of the left pinky finger (Figure 1(D,E)). At that time, an orthopaedist decided to perform amputation of the left hand. However, an ultrasound examination revealed thrombosis in the right common femoral vein, and he and his relative refused placement of a retrievable inferior vena cava filter, so the surgery was temporarily cancelled and heparin anticoagulant therapy was given. In addition, 9 days after EICU admission, the lateral side of his left lower limb had redness of local skin, and this lesion worsened, with partial ulceration and a bloody exudate appearing 14 days after EICU admission (Figure 1(F)). This lesion was gradually stabilized after daily changes of surgical dressing.

Twenty-two days after EICU admission, his respiratory function improved and mechanical ventilation was discontinued. Twenty-six days after EICU admission, his left forearm was amputated after an inferior vena cava retrievable filter placement, due to uncontrolled necrosis of the left hand (Figure 1(G,H)). Unfortunately, his condition rapidly deteriorated over the next few days, and he developed septic shock and multiple organ dysfunction syndrome (MODS). Thirty days after EICU admission he received mechanical ventilation once again, but he died of cardiac arrest 5 days later.

## Discussion

To our best knowledge, this is the second reported case of *V. anguillarum* infection worldwide, and the first reported case in China. Our patient had immune suppression due to long-term treatment with glucocorticoids because of nephrotic syndrome. Similarly, the previous report of *V. anguillarum-*associated fatal skin and soft tissue infection of left lower leg was in a female patient who was taking immunosuppressive drugs [7]. However, our case presented with a fatal skin, subcutaneous soft tissue, and muscle infection of left hand, and light skin and subcutaneous soft tissue infection of left lower limb. Unfortunately, his condition deteriorated and he ultimately died of septic shock and MODS despite administration of an intravenous antibiotic, CVVH, debridement, and fasciotomy. It is possible that the delay of the amputation of left forearm due to his initial improvement may have contributed to his death.

The two cases of *V. anguillarum* infection have another common characteristic, in that they were both related to exposure to seawater or seawater fishes. *V. anguillarum* is the oldest known fish pathogen [8], and there are 23 known serotypes (O1–O23), but only serotypes O1 and O2 (and serotype O3 to a lesser extent) can cause diseases; the other serotypes are environmental microorganisms and apparently unrelated to fish diseases [9]. Serotypes O1 and O2 of *V. anguillarum* are widely distributed, and serotype O3 only infects eels and sweetfish [9]. Therefore, we speculate that the pathogenic bacteria carried by the hairtail in our study were likely to be serotype O1 or O2 of *V. anguillarum*. The specific pathogenic mechanism of *V. anguillarum-*induced sepsis in humans is unknown. However, a study of survival, virulence-related characteristics, and transcriptomic analyses of *V. anguillarum* under starvation stress showed that a portion of the bacterial population may remain pathogenic while persisting under starvation stress by up-regulating or down-regulating multiple genes [6].

Notably, disease progression in our case was very similar to that of sepsis due to *V. vulnificus* infection [10]. Sepsis from *V. vulnificus* infection has an acute onset, an aggressive progression, and is difficult to treat [11]. About 50% to 70% of patients with sepsis from *V. vulnificus* infection die from septic shock and MODS within 48 h [12]. The infection in our case also rapidly progressed to septic shock and MODS, and our patient died 35 days after the fish bite. In contrast, the only previously reported case of *V. anguillarum*-induced sepsis survived for 3.5 days [7]. The deterioration and poor prognosis of our patient may be partly related to his compromised immunity due to the long-term use of glucocorticoids after confirmation of nephrotic syndrome. Therefore, a history of chronic disease and use of an immunosuppressant could be considered risk factors for sepsis from *V. anguillarum*. When an aquatic animal carrying *V. anguillarum* inflicts a wound on an individual, especially if the individual is immunosuppressed or otherwise vulnerable, clinicians should be alert to the possibility of wound infection, secondary sepsis, and septic shock.

However, a limitation that we did not definitely determine that the *V*. anguillarum infection was caused by the hairtail fish, because it also could have come from contaminated seawater. Nevertheless, our unique case showed that when *V. anguillarum* is present in seawater or an infected aquatic animal, it can be transmitted to humans, and can cause severe and potentially fatal disease. Even though there is only one previous report of this species being pathogenic to humans, infection of an individual who has suppressed immunity may lead to the rapid development of septic shock. The rarity of reports of human pathogenicity of *V. anguillarum* makes early diagnosis and treatment difficult. Removal of the infection source in addition to antibiotic intervention in cases with suspected *V. anguillarum*-induced sepsis is crucial in reducing the risk of mortality.

## Supplementary Material

Supplemental Material
