# Supplementary material for: Severe infection by Vibrio anguillarum following a bite by a marine fish: a case report
Source: Emerg Microbes Infect. 2023 May 5;12(1):2204145. doi: 10.1080/22221751.2023.2204145 (PMC10165925; doi:10.1080/22221751.2023.2204145)
Supplement: Supplemental Material [file TEMI_A_2204145_SM8402.docx]

**Metagenomics next-generation sequencing**

Metagenomics next-generation sequencing (mNGS) of blood and blister fluid samples was performed to identify the potential pathogens using a rapid on-site mNGS platform at the First Affiliated Hospital of Dalian Medical University. The QIAamp UCP Pathogen Mini kit (QIAGEN, Germany) was used for DNA extraction. The extracted DNA was quantified using a Qubit dsDNA HS Assay kit (Thermo Fisher, USA). The library was constructed using Nextera DNA Flex kit (Illumina, San Diego, CA, USA) according to the manufacturer’s instructions. The Qubit and Agilent 2100 Bioanalyzer (Agilent Technologies, Santa Clara, USA) were used to assess the quality of the DNA library. The qualified library was finally sequenced on a Nextseq 550 platform (Illumina, San Diego, USA) using a 50-cycle single-end sequencing strategy.

After sequencing, adaptor contamination, low-quality reads, duplicate reads, and low-complexity reads were removed from the raw data using fastp (version 0.20.0) with default parameters. Human DNA was also filtered out by mapping to the human reference genome (hg38) using Burrows-Wheeler Aligner software (version 0.7.17). The remaining reads were then aligned to the current Microbial Genome database, which covers 12,468 bacterial species (including 206 mycobacterial species), 10,061 viral taxa, 2680 fungi, and 654 parasites that are associated with human diseases. Finally, 1,554,086 standardized reads from the blister fluid sample and 1115 standardized reads from the blood sample were uniquely aligned to the *Vibrio anguillarum* genome.


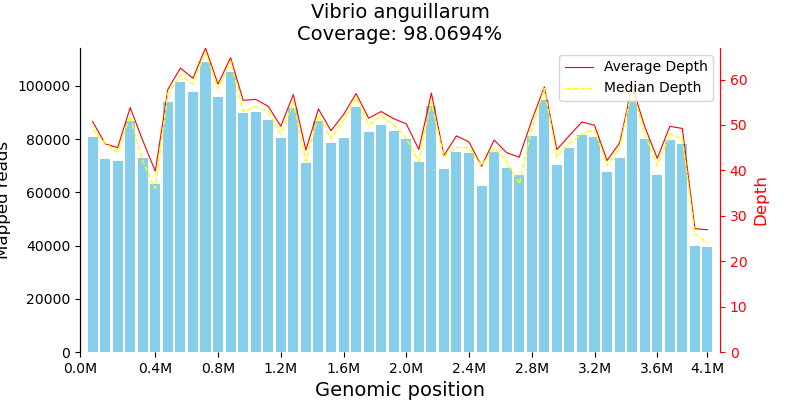


**Figure S1.** Coverage of *Vibrio anguillarum* detected by mNGS in blister fluid sample.


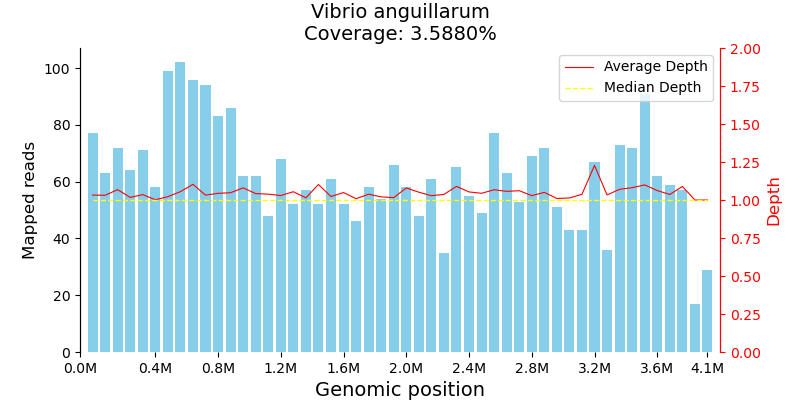


**Figure S2.** Coverage of *Vibrio anguillarum* detected by mNGS in blood sample)

**Table S1****.** High-throughput sequencing results of pathogenic microbial DNA from a blood sample.

| **Type** | **Genus** | |  | **Species** | |
| --- | --- | --- | --- | --- | --- |
|  | [**Latin Name**](javascript:;) | **Gene sequence number** |  | [**Latin Name**](javascript:;) | **Gene sequence number** |
| [**Gram-negative bacillus**](javascript:;) | *Vibrio* | 1157 |  | *Vibrio anguillarum* | 1115 |

**Table S2.** High-throughput sequencing results of pathogenic microbial DNA from a blister fluid sample.

| **Type** | **Genus** | |  | **Species** | |
| --- | --- | --- | --- | --- | --- |
|  | [**Latin Name**](javascript:;) | **Gene sequence number** |  | [**Latin Name**](javascript:;) | **Gene sequence number** |
| [**Gram-negative bacillus**](javascript:;) | *Vibrio* | 1646189 |  | *Vibrio anguillarum* | 1554086 |
